# Establishing the Roles of the Dorsal and Ventral Striatum in Humor Comprehension and Appreciation with fMRI

**DOI:** 10.1523/JNEUROSCI.1361-23.2023

**Published:** 2023-12-06

**Authors:** Margaret Prenger, Madeline Gilchrist, Kathryne Van Hedger, Ken N. Seergobin, Adrian M. Owen, Penny A. MacDonald

**Affiliations:** ^1^BrainsCAN, University of Western Ontario, London, Ontario N6A 3K7, Canada; ^2^Western Institute for Neuroscience, University of Western Ontario, London, Ontario N6A 3K7, Canada; ^3^Department of Neuroscience, University of Western Ontario, London, Ontario N6A 3K7, Canada; ^4^Clinical Neurological Sciences, Schulich School of Medicine & Dentistry, University of Western Ontario, London, Ontario N6A 3K7, Canada; ^5^Departments of Physiology & Pharmacology and Psychology, University of Western Ontario, London, Ontario N6A 3K7, Canada

**Keywords:** dorsal striatum, fMRI, humor appreciation, humor comprehension, ventral striatum

## Abstract

Humor comprehension (i.e., getting a joke) and humor appreciation (i.e., enjoying a joke) are distinct, cognitively complex processes. Functional magnetic resonance imaging (fMRI) investigations have identified several key cortical regions but have overlooked subcortical structures that have theoretical importance in humor processing. The dorsal striatum (DS) contributes to working memory, ambiguity processing, and cognitive flexibility, cognitive functions that are required to accurately recognize humorous stimuli. The ventral striatum (VS) is critical in reward processing and enjoyment. We hypothesized that the DS and VS play important roles in humor comprehension and appreciation, respectively. We investigated the engagement of these regions in these distinct processes using fMRI. Twenty-six healthy young male and female human adults completed two humor-elicitation tasks during a 3 tesla fMRI scan consisting of a traditional behavior-based joke task and a naturalistic audiovisual sitcom paradigm (i.e., *Seinfeld* viewing task). Across both humor-elicitation methods, whole-brain analyses revealed cortical activation in the inferior frontal gyrus, the middle frontal gyrus, and the middle temporal gyrus for humor comprehension, and the temporal cortex for humor appreciation. Additionally, with region of interest analyses, we specifically examined whether DS and VS activation correlated with these processes. Across both tasks, we demonstrated that humor comprehension implicates both the DS and the VS, whereas humor appreciation only engages the VS. These results establish the role of the DS in humor comprehension, which has been previously overlooked, and emphasize the role of the VS in humor processing more generally.

**SIGNIFICANCE STATEMENT** Humorous stimuli are processed by the brain in at least two distinct stages. First, humor comprehension involves understanding humorous intent through cognitive and problem-solving mechanisms. Second, humor appreciation involves enjoyment, mirth, and laughter in response to a joke. The roles of smaller subcortical brain regions in humor processing, such as the DS and VS, have been overlooked in previous investigations. However, these regions are involved in functions that support humor comprehension (e.g., working memory ambiguity resolution, and cognitive flexibility) and humor appreciation (e.g., reward processing, pleasure, and enjoyment). In this study, we used neuroimaging to demonstrate that the DS and VS play important roles in humor comprehension and appreciation, respectively, across two different humor-elicitation tasks.

## Introduction

Humor is a ubiquitous human experience that serves an adaptive purpose by facilitating social interactions. It is a higher-order ability and requires the integration of multiple cognitive processes. Humor processing can be separated into at least two distinct components—humor comprehension and humor appreciation (Ziv and Labelle, 1984).

Humor comprehension (i.e., getting the joke) is a problem-solving process in which one detects and resolves some incongruity or absurdity to reveal the joke (Suls, 1972). Humor appreciation refers to the subjective amusement or mirth experienced on realizing the joke. Although humor comprehension generally occurs only once, humor appreciation can be experienced repeatedly with further elaboration, explaining why some jokes remain funny even once the punchline is known.

Advances in neuroimaging allow researchers to explore brain regions involved in humor processing. Functional magnetic resonance imaging (fMRI) has revealed many cortical regions as integral to humor processing. Chang et al. (2023) identified blood oxygen level-dependent (BOLD) activation in the inferior frontal gyrus, the medial frontal gyrus, the superior frontal gyrus, the middle temporal gyrus, and the inferior parietal lobule in incongruity detection and resolution (i.e., humor comprehension). In contrast, activation of the ventromedial prefrontal cortex, the amygdala, the anterior insula, the nucleus accumbens, and the midbrain occurred during the elaboration stage (i.e., humor appreciation). Activation of fronto-temporoparietal areas during humor comprehension and of mesocorticolimbic areas during appreciation aligns with the conclusions of two meta-analyses of 20 and 57 fMRI humor processing studies, respectively (Vrticka et al., 2013; Farkas et al., 2021).

The role of the dorsal striatum (DS; i.e., dorsal caudate nucleus and putamen) in humor processing has generally been overlooked. Although activations of the left putamen (Iwase et al., 2002; Filik et al., 2019; Sanz-Arigita et al., 2021), the right putamen (Goldin et al., 2005; Neely et al., 2012; Shibata et al., 2014), the left caudate (Sanz-Arigita et al., 2021), and the right caudate (Goldin et al., 2005; Osaka et al., 2014; Sanz-Arigita et al., 2021) have been identified in studies of humor processing, most authors do not put importance on these findings or discuss their implications. Filik et al. (2019) were the lone authors to discuss the role of the putamen in language processing and how this could contribute to humor comprehension. DS involvement in ambiguity resolution (Crinion et al., 2006; MacDonald and Monchi, 2011; Mestres-Missé et al., 2012), suppression of prepotent responses (Zandbelt and Vink, 2010; MacDonald and Monchi, 2011; Akkermans et al., 2018), working memory (Lewis et al., 2004; MacDonald and Monchi, 2011; Darvas et al., 2014), and set shifting (MacDonald and Monchi, 2011; Darvas et al., 2014), which are essential for humor comprehension, have not been considered in the context of humor processing. The tendency is to ignore these DS activations or explain them in the context of reward processing, although experiencing humorous stimuli as rewarding pertains to humor appreciation, a process that has been shown clearly to implicate the ventral striatum (VS; nucleus accumbens and ventral caudate nucleus and putamen, z ≤ 2 using MRI; Mobbs et al., 2003; Azim et al., 2005; Mobbs et al., 2005; Watson et al., 2007; Neely et al., 2012; Noh et al., 2014; Shibata et al., 2014) and not the DS. Discounting the role if the DS in cognitive functions and misattributing all striatal activations in humor processing to affective/reward functions has caused subregions of the striatum to be excluded in reviews of the literature and theories of humor processing.

Our aim was to directly investigate the distinct contributions of the DS and VS in humor processing. We predicted that humor comprehension will involve the DS, whereas humor appreciation will engage the VS. We investigated these hypotheses using both a traditional behavior-based humor processing task and a naturalistic sitcom viewing method in fMRI with striatal regions of interest (ROIs).

## Materials and Methods

### Participants

Twenty-six young, healthy individuals participated in this study (11 male; M_age_ = 22.35, SD_age_ = 3.43; M_education_ = 16.40, SD_education_ = 2.61). All participants had normal or corrected-to-normal vision, had no history of neurologic or psychiatric disorders, and did not abuse drugs or alcohol at the time of participation. All participants provided informed consent according to the Declaration of Helsinki (World Medical Association, 2013), and all procedures were approved by the Research Ethics Board at University of Western Ontario.

### Experimental design

#### Joke task

Participants completed a humor processing task (i.e., joke task) that involved listening to 40 randomly selected audio clips of jokes from a possible bank of 80 stimuli, as well as 40 randomly selected audio clips of neutral nonjoke sentences from a possible bank of 80 stimuli while neural activity was measured using fMRI. The majority of these audio clip stimuli (92 of 160) was used in previous studies (Bekinschtein et al., 2011; Fiacconi and Owen, 2015), and joke and nonjoke stimuli were presented in random order. All audio clip stimuli were recorded in a male voice and spoken neutrally to not reveal whether the audio clip was a joke or nonjoke based on intonation or prosody. The audio was presented through MRI-compatible headphones. A short movie clip was played for participants in the scanner before the onset of the experimental task to ensure that participants could hear through both headphones and that the volume was appropriate.

**Figure 1.**
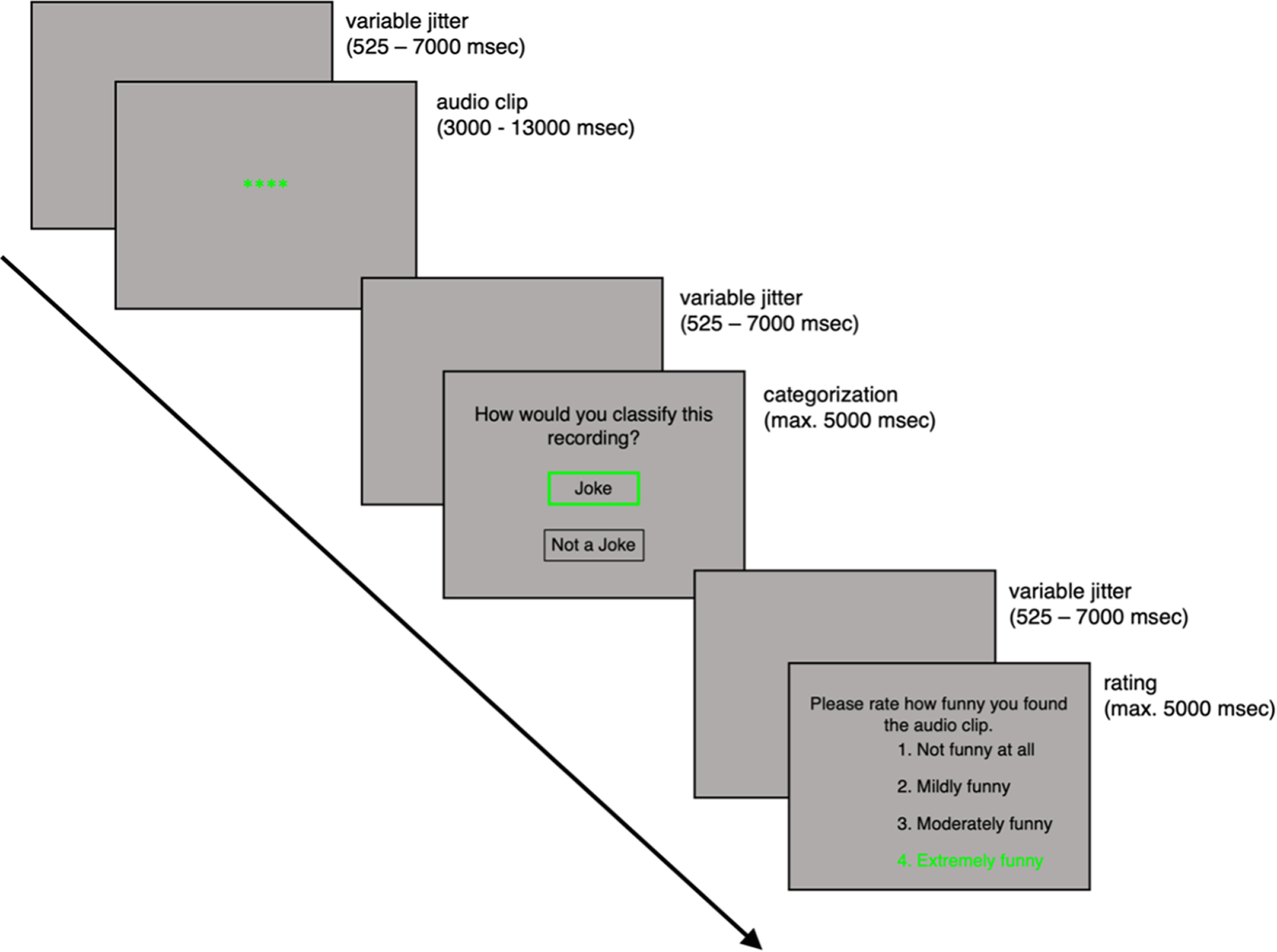
fMRI humor processing task. Participants listened to joke and nonjoke audio clips ranging from 3 to 13 s in length. Following this, they were asked to categorize the audio clip as either a joke or a nonjoke. Next, they were asked to rate how funny the audio clip was, regardless of whether it was a joke or not and regardless of their previous response. Participants used an MRI-safe button box to move their selection (in green) up or down and confirm their response. Each response screen timed out if a response was not made after 5000 ms. Intertrial and inter-response intervals were jittered with variable durations sampled from an exponential distribution (minimum = 525 ms; mean = 2500 ms; maximum = 7000 ms). max, Maximum.

Following the presentation of each audio clip, participants were asked to indicate whether they thought the audio clip was a joke, or not a joke. For all stimuli (jokes and nonjokes), they were also asked to rate how funny they found each audio clip on a scale from one (not funny at all) to four (extremely funny). Intertrial and inter-response intervals were jittered with variable durations sampled from an exponential distribution (minimum, 525 ms; mean = 2500 ms; maximum, 7000 ms). Participants used a handheld Current Designs four-button fiber optic response pad (HHSC-1×4-L) to make their responses by moving a green selection highlight up (index finger, button 2) or down (middle finger, button 3) and confirming their response (ring finger, button 4). The starting position of the green highlighted selection was randomized on each response screen to mitigate biases in response times (RTs) for selections that were closer to or farther from the starting position. Participants had a maximum of 5000 ms for each response (Fig. 1). Before completing the task, all participants watched a video containing detailed instructions of the procedure. Participants were provided an opportunity to ask questions for further clarification if necessary.

#### *Seinfeld* viewing task

Immediately following the humor processing task, participants were shown a full episode of the sitcom *Seinfeld*. Half the participants were shown the episode “The Airport” (https://www.imdb.com/title/tt0697735/). The other half of participants were shown the episode “The Movie” (https://www.imdb.com/title/tt0697735/). These specific episodes were selected because of their relative lack of overt racial and sexual humor and their focus on common activities that most individuals have previously experienced (i.e., traveling in an airplane and going to the movie theater). The episodes were visually projected onto a screen at the end of the magnet bore, which participants viewed through a mirror. Participants were instructed to watch and listen to the episode, refrain from falling asleep, and be prepared to answer questions pertaining to the video after the episode.

Following the episode and outside the scanner, participants completed a questionnaire that evaluated their prior familiarity with *Seinfeld*, how frequently they watched sitcoms and funny television in general, and how funny they found the episode of *Seinfeld*, and it included some true/false questions about the plot of the episode to ensure that they had indeed attended to the episode.

### Imaging acquisition

All imaging data were collected on a 3 tesla Siemens Magnetom Prisma Fit MRI scanner at the Center for Functional and Metabolic Mapping in the Robarts Research Institute at the University of Western Ontario. Data were acquired using a 32-channel head coil.

First, a localizer image was obtained to identify the optimal scanning area relative to the participant's head position. Separate T2*-weighted multiband echoplanar imaging (EPI) functional scans were acquired during the humor processing task and the *Seinfeld* episode with the following parameters: repetition time (TR) = 1000 ms, echo time (TE) = 30 ms, 48 slices oriented along the anterior and posterior commissure with 2.5 mm thickness, flip angle = 40°, field of view (FOV) = 220 × 220 mm^2^, voxel size = 2.5 × 2.5 × 2.5 mm^3^, and multiband factor = 4. Finally, a T1-weighted (T1w) MPRAGE anatomic scan was acquired with the following parameters: TR = 2400 ms, TE = 2.28 ms, 192 sagittal slices with 0.80 mm thickness, flip angle = 8°, FOV = 256 × 256 mm^2^, and voxel size = 0.8 × 0.8 × 0.8 mm^3^.

### Imaging preprocessing

Results included in this manuscript were achieved through image preprocessing performed using fMRIPrep 1.5.4 software (Esteban et al., 2019; https://zenodo.org/records/8206595); RRID:SCR_016216), which is based on Nipype 1.3.1 software (Gorgolewski et al., 2011; https://zenodo.org/records/6834519); RRID:SCR_002502). Visualization of fMRI data were conducted with the MRIcroGL tool (version 13.1; Rorden and Brett, 2000).

#### Anatomical data preprocessing

The T1w image was corrected for intensity nonuniformity (INU) with N4BiasFieldCorrection (Tustison et al., 2010), distributed with ANTs 2.2.0 (Advanced Normalization Tools; Avants et al., 2008; RRID:SCR_004757) software, and used as T1w reference throughout the workflow. The T1w reference was then skull stripped with a Nipype implementation of the antsBrainExtraction.sh workflow (from ANTs), using OASIS30ANTs as the target template. Brain tissue segmentation of CSF, white-matter (WM) and gray-matter (GM) was performed on the brain-extracted T1w using Functional MRI of the Brain (FMRIB) Software Library (FSL; version 5.0.9; RRID:SCR_002823; Zhang et al., 2001) Automated Segmentation Tool. Brain surfaces were reconstructed using the recon-all command (FreeSurfer 6.0.1; RRID:SCR_001847; Dale et al., 1999), and the brain mask estimated previously was refined with a custom variation of the method to reconcile ANTs-derived and FreeSurfer-derived segmentations of the cortical gray matter of Mindboggle (RRID:SCR_002438; Klein et al., 2017). Volume-based spatial normalization to one standard space (MNI152NLin2009cAsym) was performed through nonlinear registration with the antsRegistration function (ANTs 2.2.0), using brain-extracted versions of both T1w reference and the T1w template. The template selected for spatial normalization was ICBM 152 Nonlinear atlases version 2009 (Fonov et al., 2009; RRID:SCR_008796; TemplateFlow ID, MNI152NLin2009cAsym).

#### Functional data preprocessing

For each of the BOLD runs found per subject (across both tasks), the following preprocessing was performed. First, a reference volume and its skull-stripped version were generated using a custom methodology of fMRIPrep. A B0-nonuniformity map (or fieldmap) was estimated based on a phase-difference map calculated with a dual-echo GRE (gradient-recall echo) sequence, processed with a custom workflow of SDCFlows inspired by the epidewarp.fsl script and further improvements in HCP (Human Connectome Project) Pipelines (Glasser et al., 2013). The fieldmap was then coregistered to the target EPI reference run and converted to a displacements field map (amenable to registration tools such as ANTs) with FSL FUGUE (FMRIB Utility for Geometrically Unwarping EPIs) and other SDCflows tools. Based on the estimated susceptibility distortion, a corrected EPI reference was calculated for a more accurate coregistration with the anatomic reference. The BOLD reference was then coregistered to the T1w reference using bbregister (FreeSurfer), which implements boundary-based registration (Greve and Fischl, 2009). Coregistration was configured with six degrees of freedom. Head-motion parameters with respect to the BOLD reference (transformation matrices and six corresponding rotation and translation parameters) are estimated before any spatiotemporal filtering using MCFLIRT (FMRIB Linear Image Registration Tool with motion correction; FSL 5.0.9, Jenkinson et al., 2002). BOLD runs were slice-time corrected using 3dTshift from AFNI (Analysis of Functional NeuroImages) 20160207 (Cox and Hyde, 1997; RRID:SCR_005927). The BOLD time series were resampled to surfaces in the fsaverage5 spaces. The BOLD time series (including slice-timing correction when applied) were resampled onto their original native space by applying a single composite transform to correct for head motion and susceptibility distortions. These resampled BOLD time series are referred to as “preprocessed BOLD in original space,” or just “preprocessed BOLD.” The BOLD time-series were resampled into standard space, generating a preprocessed BOLD run in ['MNI152NLin2009cAsym'] space. First, a reference volume and its skull-stripped version were generated using a custom methodology of fMRIPrep. Several confounding time series were calculated based on the preprocessed BOLD, that is, framewise displacement (FD), DVARS (D refers to a derivative of fMRI time course, VARS refers to root-mean-squared variance), and three region-wise global signals. FD and DVARS are calculated for each functional run, both using their implementations in Nipype (following the definitions by Power et al., 2014). The three global signals are extracted within the CSF, the WM, and the whole-brain masks. Additionally, a set of physiological regressors was extracted to allow for component-based noise correction (CompCor; Behzadi et al., 2007). Principal components are estimated after high-pass filtering the preprocessed BOLD time-series (using a discrete cosine filter with 128 s cutoff) for the two CompCor variants, temporal (tCompCor) and anatomic (aCompCor). tCompCor components are then calculated from the top 5% variable voxels within a mask covering the subcortical regions. This subcortical mask is obtained by heavily eroding the brain mask, which ensures it does not include cortical GM regions. For aCompCor, components are calculated within the intersection of the aforementioned mask, and the union of CSF and WM masks are calculated in T1w space after their projection to the native space of each functional run (using the inverse BOLD-to-T1w transformation). Components are also calculated separately within the WM and CSF masks. For each CompCor decomposition, the *k* components with the largest singular values are retained such that the time series of the retained components are sufficient to explain 50% of variance across the nuisance mask (CSF, WM, combined, or temporal). The remaining components are dropped from consideration. The head motion estimates calculated in the correction step were also placed within the corresponding confounds file. The confound time series derived from head motion estimates and global signals were expanded with the inclusion of temporal derivatives and quadratic terms for each (Satterthwaite et al., 2013). Frames that exceeded a threshold of 0.5 mm FD or 1.5 standardized DVARS were annotated as motion outliers. All resamplings can be performed with a single interpolation step by composing all the pertinent transformations (i.e., head motion transform matrices, susceptibility distortion correction when available, and coregistrations to anatomic and output spaces). Gridded volumetric resamplings were performed using antsApplyTransforms (ANTs), configured with Lanczos interpolation to minimize the smoothing effects of other kernels (Lanczos, 1964). Nongridded surface resamplings were performed using the mri_vol2surf command (FreeSurfer). Many internal operations of fMRIPrep use the NiLearn 0.6.0 (Abraham et al., 2014; RRID:SCR_001362) software package, mostly within the functional processing workflow. For more details of the pipeline, see the fMRIPrep documentation (the section corresponding to workflows). Following this preprocessing pipeline, the normalized data were spatially smoothed with an 8 mm full-width half-maximum Gaussian kernel using SPM12 (Statistical Parametric Mapping 12; Wellcome Centre for Human Neuroimaging) software.

### Statistical analysis

Demographic and behavioral data were analyzed with R statistical computing software (version 4.2.0; https://www.R-project.org/) and R Studio (version 2022.07.01; http://www.rstudio.com/). Data were examined for outliers above or below 3× the interquartile range. RT data for both humor comprehension and appreciation were also examined for time-out instances in which participants failed to respond within the 5 s time limit.

#### Imaging analysis

fMRI data were analyzed using SPM12 and MATLAB (version R2022a, MathWorks).

##### Joke task fMRI analysis

Separate first-level, fixed-effects analyses were performed for each individual participant. For humor comprehension, a general linear model (GLM) was constructed in which the canonical hemodynamic response function was convolved with the onsets and durations of the auditory stimuli for each stimulus category. Only the trials in which the participant correctly identified the stimulus as either a joke or a nonjoke were included in this GLM. Two regressors of interest were included in the model, Jokes and Nonjokes. Average CSF signal, global signal, and the six head motion parameters (translation and rotation in *x*, *y*, and z dimensions) were included as covariates of no interest. Following model estimation, a single contrast of interest was examined, the main effect of Joke (i.e., Joke > Nonjoke). For humor appreciation, a separate GLM was constructed in which two regressors of interest were modeled, Funny (i.e., trials in which participants' funniness ratings equaled or exceeded 2), and Not Funny (i.e., trials in which that participants' funniness ratings equaled 1), along with average CSF, global signal, and the six head motion parameters as covariates of no interest. All trials were analyzed. A single contrast of interest was examined, the main effect of Funny (Funny > Not Funny).

Next, second-level random effects analyses were conducted. Contrast images from each participant were examined in separate group-level *t* tests for each main and interaction effect. Consistent with previous humor processing literature, whole-brain analyses were examined using a conservative voxel-level FWE-corrected height threshold of *p* < 0.05 and a cluster-level extent threshold of *k* = 10 consecutive voxels. The anatomic location of the peak voxel within each cluster that survived this threshold was identified using the automated anatomic labeling atlas 3 (AAL3; Rolls et al., 2020).

**Figure 2.**
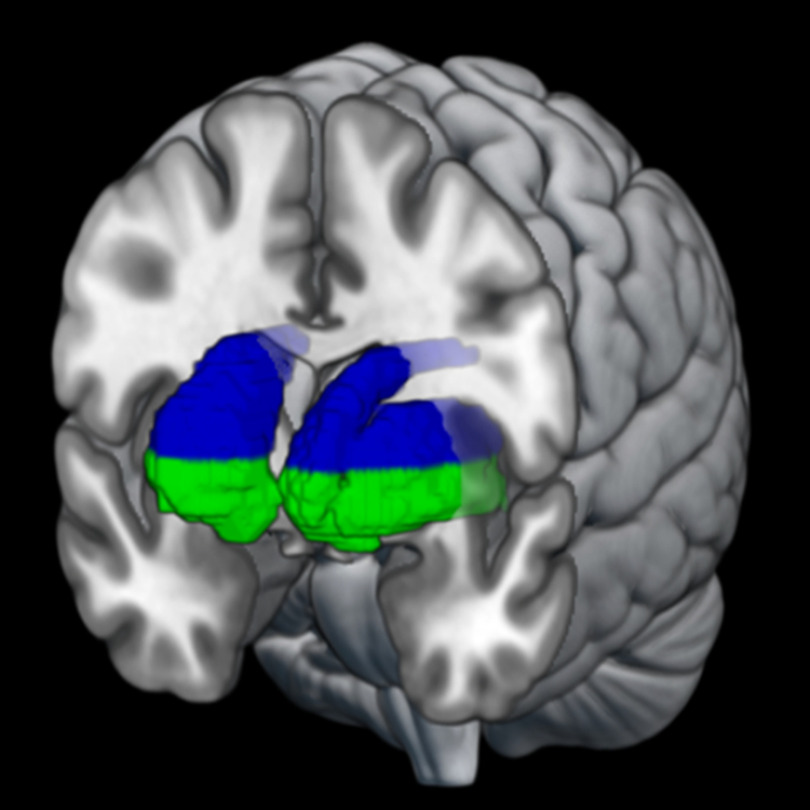
Dorsal (blue) and ventral (green) striatum regions of interest, delineated by *z* = 2 mm in MNI space.

To specifically test our hypothesis that the striatum is involved in humor processing, ROIs were generated using the MarsBaR toolbox (Brett et al., 2002) based on those described in Hiebert et al. (2019). Briefly, the DS ROI contained the bilateral dorsal caudate nucleus and the bilateral dorsal putamen at a level of z > 2 mm in MNI space. The VS ROI contained the bilateral nucleus accumbens, bilateral ventral caudate nucleus, and bilateral ventral putamen at a level of z ≤ 2 mm in MNI space. The *z* = 2 mm cutoff was based on a review by Postuma and Dagher (2006). These ROIs are depicted in MNI space in Figure 2. For each contrast of interest, average beta values for DS and VS ROIs were estimated and compared with zero with Bonferroni-corrected one-sample *t* tests. In the case of nonsignificant results, support in favor of the null hypothesis was examined with Bayesian analysis (Dienes, 2014; Keysers et al., 2020). The magnitude of the resulting Bayes factor (BF_10_), a ratio of evidence for or against a null hypothesis, was evaluated compared with the cutoffs suggested by Jeffreys (1939), in which a BF_10_ > 3 represents substantial evidence in favor of the alternative hypothesis.

##### *Seinfeld* viewing task fMRI analysis

**Figure 3.**
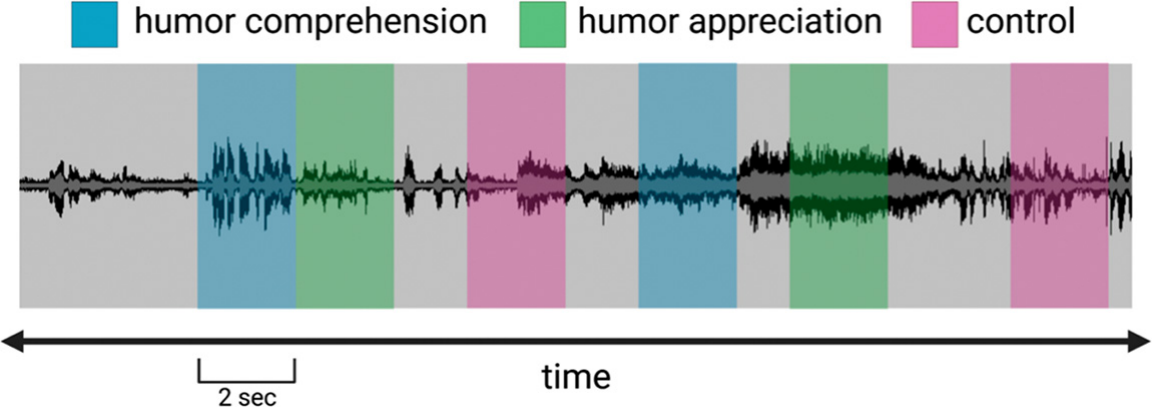
*Seinfeld* viewing task events are shown on a representative waveform. Humor comprehension events were defined as the 2 s before the onset of laughter in the episode laugh track (blue). Humor appreciation events were defined as the middle 2 s of laughter in the laugh track (green). Control events were defined as the 2 s epochs directly between neighboring comprehension and appreciation events (magenta).

Our *Seinfeld* viewing paradigm was modeled on the study of Moran et al. (2004). As shown in Figure 3, the laugh track of each episode was used to create event epochs for (a) humor comprehension, defined as the 2 s before the onset of the laugh track; (b) humor appreciation, specified as the middle 2 s of laughter in the laugh track, and (c) control, characterized as the 2 s period occurring midway between the end of the last humor appreciation event and the next humor comprehension event. Laugh track epochs were identified by four independent raters. The resulting event onsets and durations for each condition were based on the consensus of these raters and confirmed by visual inspection of the audio waveform of the episode.

As opposed to the Joke task, which used canned jokes that are rarely encountered in everyday life, the *Seinfeld* viewing task represents a uniquely naturalistic approach to evaluating humor comprehension and appreciation. However, it is important to note that the humor comprehension and appreciation events in the *Seinfeld* viewing task are not participant driven like those of the Joke task are. The humor comprehension and appreciation events in the *Seinfeld* viewing task are based on the moments of the episode(s) that a live studio audience from the 1990s found to be funny, which might not necessarily represent the moments of the episode(s) that our participants found to be funny.

First-level fixed-effects analyses were performed for each individual participant. For humor comprehension, a GLM was constructed, convolving the canonical hemodynamic response function with the onsets and durations of the comprehension and control events. These events were used as regressors of interest along with the CSF signal, global signal, and six head motion parameters as covariates of no interest. Following model estimation, a single contrast of interest was examined, that is, the main effect of humor comprehension (i.e., humor comprehension > control). For humor appreciation, a separate GLM was constructed, in which a single contrast of interest was examined, that is, the main effect of humor appreciation (i.e., humor appreciation > control). We considered activations during this contrast to be attributable to the experience of humor appreciation as opposed to a reaction to hearing others laugh, given that Moran et al. (2004) observed nearly identical activations for humor appreciation across their two separate sitcom-viewing fMRI experiments that did and did not include a laugh track.

Next, second-level random effects analyses were conducted. Contrast images from each participant were examined in separate group-level *t* tests for each main and interaction effect. Consistent with the *Seinfeld* viewing study by Moran et al. (2004), whole-brain analyses were examined. However, we used a more conservative voxel-level FWE-corrected height threshold of *p* < 0.05 and a cluster-level extent threshold of *k* = 10 consecutive voxels. The anatomic location of the peak voxel within each cluster that survived this threshold was identified using the AAL3 (Rolls et al., 2020). To specifically test our hypothesis that the striatum is involved in humor processing, we used the same DS and VS ROIs as described above. For each contrast of interest, average beta values for DS and VS ROIs were estimated and included in the analyses.

#### Conjunction analysis

To determine which regions are jointly activated during our contrasts of interest between the Joke task and the *Seinfeld* viewing task for humor comprehension and humor appreciation, we conducted conjunction analyses using the procedure suggested by Nichols et al. (2005).

## Results

### Behavioral results

#### Joke task: humor comprehension

Humor comprehension was calculated as the percentage of correctly identified joke and nonjoke stimuli. These data were entered into a paired *t* test. The difference in humor comprehension accuracy between jokes and nonjokes did not reach significance (*t*_(51)_ = −1.87, *p* = 0.068), with Joke stimuli (mean = 86.06%, 95% CI [82.22, 89.89]) being slightly more accurately categorized than Nonjoke stimuli (mean = 81.35%, 95% CI [82.22, 89.89]).

#### Joke task: humor appreciation

Humor appreciation was calculated as the average funniness ratings of joke and nonjoke stimuli. These data were entered into a paired *t* test. Unsurprisingly, there was a significant difference in funniness estimates between jokes and nonjokes (*t*_(51)_ = −18.54, *p* < 0.001). There was also a significant positive correlation between funniness ratings for joke stimuli and the Sense of Humor Questionnaire 6 scores (*r* = 0.29, *p* = 0.03), suggesting that participants with a greater sense of humor tended to rate joke stimuli as funnier.

#### *Seinfeld* viewing task: postscan questionnaire

A chi-square test of independence was conducted to examine the proportion of individuals in each episode group who had never watched a single episode of *Seinfeld* to those who had prior experience with the show. There was not a significant difference in the familiarity with *Seinfeld* (χ^2^ = 0.62, *p* = 0.43) between participants assigned to the different *Seinfeld* episodes. Finally, a two-sample *t* test evaluated the difference in the mean funniness rating accorded to each episode of *Seinfeld* by the participants who watched “The Airport” and “The Movie,” respectively. There was no significant difference in funniness ratings between the episodes (*t*_(24)_ = −1.17, *p* = 0.26). Together, the two episodes of *Seinfeld* and the groups of participants who viewed each episode were deemed equivalent. All subsequent analyses were conducted on data collapsed across the groups of participants who viewed different episodes of *Seinfeld*. All postscanning questionnaire data are shown in Table 1.

**Table 1.** *Seinfeld*-viewing task postscan questionnaire data

Postscan questionnaire item	The Airport (*n* = 13)	*The Movie* (*n* = 13)	Overall (*N* = 26)
How familiar are you with *Seinfeld*?			
I had never watched an episode of *Seinfeld* previously	5 (38%)	6 (46%)	11 (42%)
I had watched a few episodes here and there, but never a full season	8 (62%)	3 (23%)	11 (42%)
I have watched at least one season but not the entire series	0 (0%)	2 (15%)	2 (8%)
I have watched the entire series once	0 (0%)	1 (8%)	1 (4%)
I have watched the entire series multiple times	0 (0%)	1 (8%)	1 (4%)
Average number of days since last watching *Seinfeld*?^†^	497.63 (±607.13)	505.57 (±695.32)	501.33 (±625.72)
How frequently do you watch sitcoms, in general? (no. of episodes per month)			
Never (0 per month)	2 (15%)	4 (31%)	6 (23%)
Sometimes (1–5 per month)	4 (31%)	3 (23%)	7 (27%)
Often (5–10 per month)	2 (15%)	1 (8%)	3 (12%)
Very often (10–15 per month)	0 (0%)	0 (0%)	0 (0%)
Always (15+ per month)	5 (38%)	5 (38%)	10 (38%)
Have you ever seen this episode of *Seinfeld* before?			
No	13 (100%)	12 (92%)	25 (96%)
Yes	0 (0%)	1 (8%)	1 (4%)
How funny was this episode of *Seinfeld*, from 1 (not funny at all) to 10 (funniest thing I've seen in my life)?^†^	4.46 (±2.15)	5.31 (±1.49)	4.88 (±1.86)

Data are presented as absolute values and percentage of sample in parentheses except where indicated by a dagger (†), which specifies that data represent the mean and standard deviation.

### Whole-brain fMRI results

#### Joke task: humor comprehension

Significant activations for the contrast of interest pertaining to humor comprehension (Joke > Nonjoke) are listed in Table 2. Only trials in which stimuli were correctly categorized as either jokes or nonjokes were analyzed. Clusters that contain striatal or midbrain structures are marked with a dagger in Table 2.

**Table 2.** Whole-brain BOLD activation in the joke task for humor comprehension (Joke > Nonjoke contrast)

Anatomical region	Coordinates (*x*, *y*, *z*)	Cluster size (*k_E_*)	*t* Value	*p*_FWE-corr_
R middle temporal gyrus (BA 21)	49, −30, −3	215	12.71	<0.001
L temporal pole (BA 38)	−48, 13, −30	114	9.93	<0.001
L middle frontal gyrus (BA 8)	−34, 16, 50	113	9.52	<0.001
L angular gyrus	−51, −60, 30	414	8.92	<0.001
L inferior frontal gyrus (pars triangularis)	−51, 18, 17	223	8.83	<0.001
L middle temporal gyrus	−61, −52, 10	319	8.74	<0.001
L putamen^†^	−18, 6, −10	82	8.74	<0.001
L superior frontal gyrus	−6, 43, 47	100	8.36	0.001
R temporal pole	49, 13, −33	93	8.29	0.001
L supplementary motor area (BA 6)	−8, 13, 67	150	7.97	0.001
L midbrain^†^	−4, −27, 2	112	7.89	0.001
L inferior frontal gyrus (pars triangularis; BA 10)	−51, 46, 0	30	7.56	0.003
L thalamus	−4, −12, 4	31	7.44	0.004

MNI coordinates, *t* values, and *p* values represent those of the peak voxel within each cluster, defined by a voxel-level FWE-corrected height threshold of *p* < 0.05 and a cluster-level extent threshold of *k* = 10. Anatomical regions represent the location of the peak voxel, identified using the automated anatomic labeling atlas version 3 (AAL3). Clusters that include striatal or midbrain structures are indicated by a dagger (†). R, Right; L, left.

#### Joke task: humor appreciation

There were no significant differences in head motion during funny and not funny trials, as evaluated by Bonferroni-corrected paired *t* tests for each of the six head motion parameters. Significant activations for the humor appreciation contrast are listed in Table 3. Again, clusters that contain striatal or midbrain structures are marked with a dagger in Table 3.

**Table 3.** Whole-brain BOLD activation in the joke task for humor appreciation (funny > not funny contrast)

Anatomical region	Coordinates (*x*, *y*, *z*)	Cluster size (*k_E_*)	*t* Value	*p*_FWE-corr_
L temporal pole (BA 38)	−51, 13, −28	77	9.12	<0.001
L middle frontal gyrus	−38, 18, 44	52	7.93	0.001
R temporal pole	54, 10, −23	31	7.41	0.003
L angular gyrus	−54, −60, 32	154	7.41	0.004
L thalamus	−1, −14, 4	13	7.25	0.005
R middle temporal gyrus	49, −32, −6	29	7.25	0.005
L superior frontal gyrus	−8, 28, 57	10	6.89	0.011
L middle temporal gyrus	−58, −30, −6	15	6.49	0.025

MNI coordinates, *t* values, and *p* values represent those of the peak voxel within each cluster, defined by a voxel-level FWE-corrected height threshold of *p* < 0.05 and a cluster-level extent threshold of *k* = 10. Anatomical regions represent the location of the peak voxel, identified using the AAL3. Clusters that include striatal or midbrain structures are indicated by a dagger (†). R, Right; L, left.

#### *Seinfeld* viewing task: humor comprehension

Moments of humor comprehension were defined as the 2 s epochs immediately preceding the onset of laughter in the laugh track of *Seinfeld* and were contrasted to 2 s control epochs selected from the midpoint between the offset and onset of consecutive laugh track epochs. Significant activations for the humor comprehension contrast are listed in Table 4. Clusters that contain striatal or midbrain structures are marked with a dagger in Table 4.

**Table 4.** Whole-brain BOLD activation in the *Seinfeld* viewing task for humor comprehension

Anatomical region	Coordinates (*x*, *y*, *z*)	Cluster size (*k_E_*)	*t* Value	*p*_FWE-corr_
R middle temporal gyrus (BA 37)	49, −72, 2	399	10.47	<0.001
L hippocampus	−31, −10, −13	367	10.12	<0.001
R temporal pole (BA 38)	32, 18, −33	145	8.93	<0.001
L middle temporal gyrus	−51, −70, 7	100	8.26	0.001
R supramarginal gyrus	64, −27, 30	80	7.71	0.002
R superior temporal gyrus	49, −42, 12	61	7.67	0.003
R amygdala	22, −4, −16	66	7.64	0.003
L fusiform gyrus	−41, −52, −18	14	7.60	0.003
R insula	36, 8, 2	36	7.57	0.003
L midbrain^†^	−1, −37, −3	12	7.48	0.004
L inferior frontal gyrus (pars orbicularis)	−44, 28, −3	24	7.45	0.004
L insula	−38, 6, 2	13	7.17	0.007
R middle frontal gyrus (BA 6)	42, 0, 54	24	7.14	0.007
R supplementary motor area	2, 16, 62	29	6.85	0.013

MNI coordinates, *t* values, and *p* values represent those of the peak voxel within each cluster, defined by a voxel-level FWE-corrected height threshold of *p* < 0.05 and a cluster-level extent threshold of *k* = 10. Anatomical regions represent the location of the peak voxel, identified using the AAL3. Clusters that include striatal or midbrain structures are indicated by a dagger (†). R, Right; L, left.

#### *Seinfeld* viewing task: humor appreciation

Moments of humor appreciation were defined as the middle 2 s of laughter in the laugh track of *Seinfeld* and were contrasted to the same 2 s control epochs selected from the midpoint of consecutive laugh track epochs, as described above. For each of the six head motion parameters, Bonferroni-corrected paired *t* tests were conducted to compare motion during humor appreciation events to control events. Importantly, none were significant, suggesting there was no difference in the amount of head motion during laugh track epochs compared with the remainder of the episode. Significant activations for the humor appreciation contrast are listed in Table 5. Clusters that contain striatal or midbrain structures are marked with a dagger in Table 5.

**Table 5.** Whole-brain BOLD activation in the *Seinfeld* viewing task for humor appreciation

Anatomical region	Coordinates (*x*, *y*, *z*)	Cluster size (*k_E_*)	*t* Value	*p*_FWE-corr_
R inferior temporal gyrus (BA 37)	52, −74, −3	2321	11.16	<0.001
L fusiform gyrus	−28, −64, −8	256	10.23	<0.001
L precuneus	−14, −47, 52	25	8.18	0.001
L cerebellum (lobule VI)	−8, −67, −8	30	7.94	0.001
R superior parietal lobule (BA 7)	26, −60, 52	40	7.65	0.003
R supramarginal gyrus	62, −34, 32	28	6.94	0.012
R precuneus (BA 7)	19, −74, 40	24	6.86	0.013

MNI coordinates, *t* values, and *p* values represent those of the peak voxel within each cluster, defined by a voxel-level FWE-corrected height threshold of *p* < 0.05 and a cluster-level extent threshold of *k* = 10. Anatomical regions represent the location of the peak voxel, identified using the AAL3. Clusters that include striatal or midbrain structures are indicated by a dagger (†). R, Right; L, left.

#### Conjunction analyses

**Figure 4.**
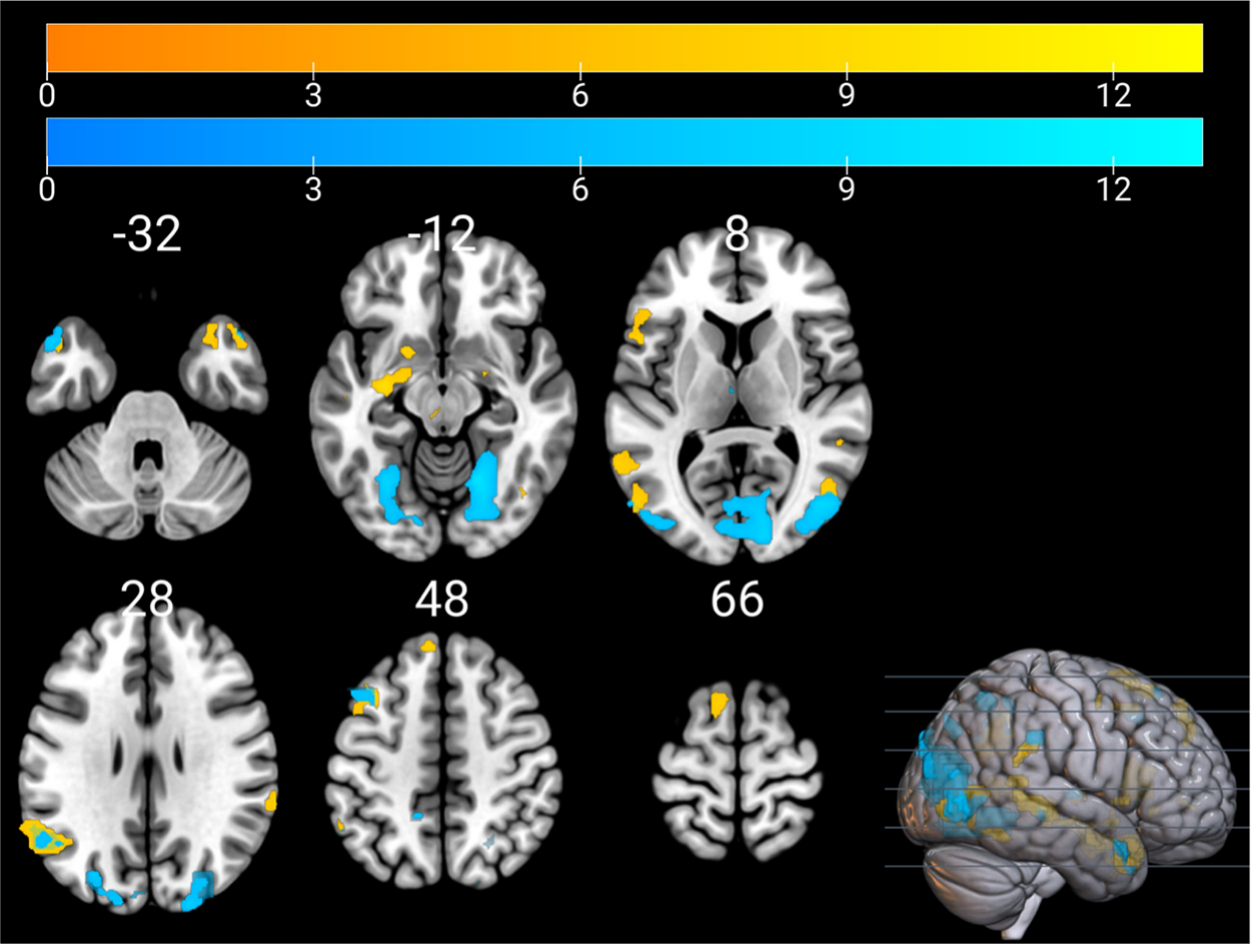
Conjunction analysis for humor comprehension (warm colors) and appreciation (cool colors) contrasts across tasks. Color bars represent *t* values.

Brain regions that were significantly activated across tasks for the humor comprehension and humor appreciation contrasts were identified using conjunction analyses. For the humor comprehension contrasts, regions that were significantly activated across tasks included the left inferior frontal gyrus, the bilateral middle frontal gyrus, the bilateral middle temporal gyrus, the bilateral temporal poles, the right fusiform gyrus, the left supplementary motor area, the left angular gyrus and the right supramarginal gyrus (i.e., the inferior parietal lobule), the left insula, the right red nucleus, the left thalamus, and the bilateral amygdala. Regions that were commonly activated by the humor appreciation contrasts across tasks included the left middle frontal gyrus, the left superior frontal gyrus, the bilateral middle temporal gyrus, the bilateral temporal poles, the right superior parietal lobule, the left angular gyrus, the right supramarginal gyrus, the left precuneus, the left lingual gyrus, the right cuneus, and the left thalamus. Activation maps of both conjunction analyses are shown in Figure 4.

### Striatal ROI results

Significant activations in the DS and VS ROIs are presented at a level of *p* < 0.05, corrected for multiple comparisons with the Bonferroni method.

#### Joke task: humor comprehension

**Figure 5.**
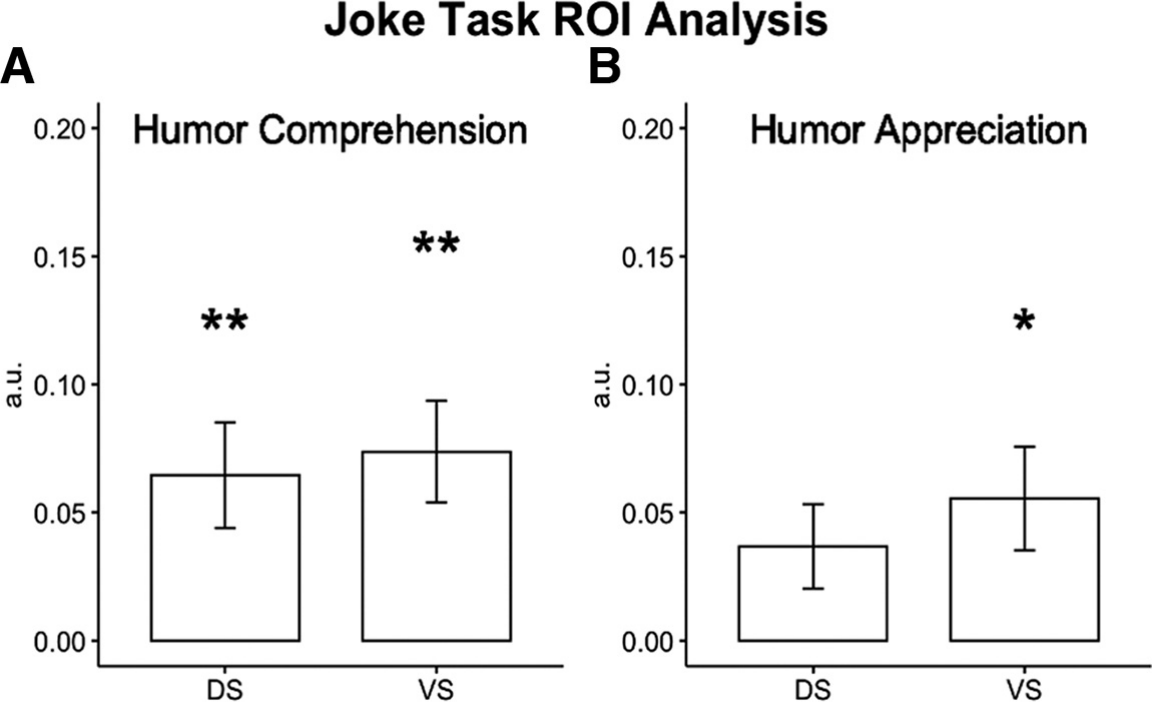
***A***, Significant activation was observed in the DS and VS during the Joke > Nonjoke contrast for correct trials only, a measure of humor comprehension. ***B***, Significant activation was observed in the VS but not the DS during the Funny > Not Funny contrast for all trials, a measure of humor appreciation. a.u., Arbitrary units. *p < 0.05, **p < 0.01.

As we did previously in the whole-brain analyses, humor comprehension was evaluated in the Joke task by the Joke > Nonjoke contrast for correct trials only. To determine whether the DS and/or VS contribute to humor comprehension, average activation during this contrast in the DS and VS ROIs was compared with zero using separate one-sample *t* tests, corrected for multiple comparisons. We observed activation that was significantly different from zero in both the DS (*t*_(25)_ = 2.94, *p* = 0.014) and the VS (*t*_(25)_ = 3.99, *p* = 0.001) ROIs (Fig. 5*A*) during humor comprehension in the Joke task.

#### Joke task: humor appreciation

For humor appreciation, we evaluated average activation in the DS and VS ROIs for the Funny > Not Funny contrast (calculated for all trials). This activation was compared with zero in separate one-sample *t* tests for each ROI. We observed significant activation in the VS (*t*_(25)_ = 3.00, *p* = 0.012), but not in the DS (*t*_(25)_ = 2.04, *p* = 0.10; Fig. 5*B*) for humor appreciation. Evaluation of this null effect using a Bayesian one-sample *t* test with default effect size priors (Cauchy scale, 0.707) suggested that there was a lack of evidence supporting DS activation during moments of humor appreciation (BF_10_ = 1.22).

#### *Seinfeld* viewing task: humor comprehension

**Figure 6.**
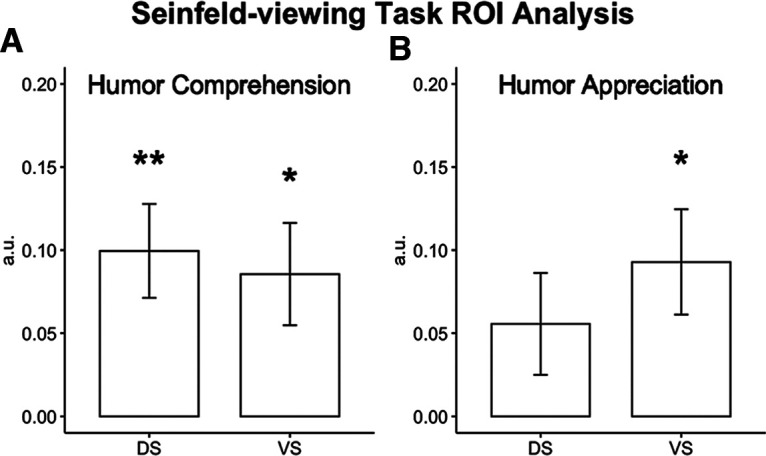
***A***, Significant activation was observed in the DS and VS during the Joke > Nonjoke contrast for correct trials only, a measure of humor comprehension. ***B***, Significant activation was observed in the VS but not the DS during the Funny > Not Funny contrast for all trials, a measure of humor appreciation. a.u., Arbitrary units. *p < 0.05, **p < 0.01.

For humor comprehension in the *Seinfeld* viewing task, we calculated the average activation in the DS and VS during the 2 s before the onset of laughter in the episode laugh track, compared with control epochs of equal duration sampled from the rest of the episode. This activation was compared with zero with separate one-sample *t* tests, corrected for multiple comparisons. We observed activation that was significantly different from zero in both the DS (*t*_(25)_ = 3.53, *p* = 0.003) and the VS (*t*_(25)_ = 2.79, *p* = 0.02) ROIs (Fig. 6*A*).

#### *Seinfeld* viewing task: humor appreciation

For humor appreciation in the *Seinfeld* viewing task, we calculated the average activation in the DS and VS during the middle 2 s of laugh-track laughter, compared with control epochs of equal duration selected from the remainder of the episode. This activation was compared with zero with separate one-sample *t* tests for each ROI, corrected for multiple comparisons. We observed activation that was significantly different from zero in the VS (*t*_(25)_ = 2.94, *p* = 0.014) ROI but not in the DS (*t*_(25)_ = 1.81, *p* = 0.016), as shown in Figure 6*B*. To evaluate the strength of evidence for the hypothesis that the DS is activated during humor appreciation, a Bayesian one-sample *t* test was conducted using default effect size priors (Cauchy scale, 0.707). There was no support for this alternative hypothesis (BF_10_ = 0.86), suggesting that the DS does not play a role in humor appreciation.

## Discussion

Using fMRI and two independent measures of humor processing, performed by the same healthy young participants, we investigated BOLD activity associated with humor comprehension and humor appreciation. In whole-brain analyses, for both tasks we found significant activation of the inferior frontal gyrus, the middle frontal gyrus, the supplementary motor area, the middle temporal gyrus, the temporal poles, and the midbrain for humor comprehension. We found common activations in the temporal cortex [i.e., Brodmann area (BA) 37 and BA 38] for humor appreciation in both tasks. In addition to whole-brain analyses, we examined BOLD signal in the DS and VS associated with humor comprehension and appreciation with an ROI approach. In both tasks, we found that humor comprehension seems to implicate both the DS and VS, whereas humor appreciation preferentially engages the VS. These findings align with our expectations that different brain regions underlie humor comprehension and appreciation and that the striatum is involved in humor processing.

Our whole-brain and conjunction analyses corroborated the findings of previous studies regarding cortical regions that are involved in humor comprehension and appreciation. For the humor comprehension contrast in the Joke task, we found significant activation in the left inferior frontal gyrus, the left middle frontal gyrus, the left superior frontal gyrus, the bilateral middle temporal gyrus, the bilateral temporal pole, the left angular gyrus, the left supplementary motor area, the left precentral gyrus, the left putamen, the left midbrain, the left thalamus, and the right amygdala. We corroborated these results with our *Seinfeld* viewing task, albeit with a slight shift in hemispheric lateralization, finding significant clusters of activation in the left inferior frontal gyrus, the right middle frontal gyrus, the bilateral middle temporal gyrus, the right superior temporal gyrus, the right temporal pole, the right supramarginal gyrus, the left fusiform gyrus, the right supplementary motor area, the bilateral insula, the left hippocampus, the left midbrain, and the right amygdala. Many of these cortical regions (e.g., the inferior frontal gyrus, the middle temporal gyrus) have been identified in previous studies of humor comprehension (Bartolo et al., 2000; Goel and Dolan, 2001; Wild et al., 2006; Samson et al., 2008, 2009; Bekinschtein et al., 2011; Chan et al., 2013; Vrticka et al., 2013; Osaka et al., 2014). The shift in hemispheric lateralization between the Joke task and *Seinfeld* viewing might be because of the differences in humor modality between these tasks. Verbal humor, which was measured in the Joke task, is associated with greater activation in the left hemisphere, whereas visual/situational humor as assessed in the *Seinfeld* viewing task, is associated with greater activation in the right hemisphere (Moran et al., 2004; Vrticka et al., 2013). We also found activation in a cluster encompassing the left putamen in this contrast, which is consistent with a previous meta-analysis of 28 studies that identified coactivation of the left anterior putamen and cortical regions such as the inferior frontal gyrus and precentral gyrus during language processing tasks (Viñas-Guasch and Wu, 2017). Finally, we also observed significant activation of the left midbrain for humor comprehension in both tasks, which, coupled with our striatal ROI findings, could indicate that humor comprehension involves dopamine signaling. For humor appreciation, we found activations of BA 38 (temporal pole) in the Joke task and BA 37 (inferior temporal gyrus) in the *Seinfeld* viewing task. Our conjunction analysis confirmed that temporal regions, among others, were activated by humor appreciation across both tasks. The temporal cortex has been implicated in laughter associated with mirth (Swash, 1972; Satow et al., 2003; Wildgruber et al., 2013; Caruana et al., 2015; Yamao et al., 2015) as opposed to nonmirthful laughter, which implicates the anterior cingulate cortex (Caruana et al., 2015), a region that was not identified in our whole-brain analyses of humor appreciation. Importantly, the temporal cortex has been identified in previous fMRI studies of humor appreciation (Mobbs et al., 2003; Kipman et al., 2012; Amir et al., 2015). Interestingly, our humor appreciation conjunction analysis also revealed activation of medial occipital regions (i.e., the lingual gyrus and the cuneus). These regions have been implicated in nonvisual functions such as language processing (Palejwala et al., 2021).

Consistent with other studies of humor processing, our whole-brain analyses showed sparse subcortical activation. Although whole-brain analysis is a popular approach for analyzing fMRI data, the height and extent thresholds that are routinely applied to correct for multiple comparisons favor larger cortical regions, making it difficult for activation in small brain regions (e.g., DS and VS) to survive these corrections. Illustrating this, most of our striatal and midbrain clusters barely exceed 10 contiguous voxels, with our largest measuring only 112 voxels in extent. Failing to account for these small-volume regions either through ROI analyses or small-volume correction might have led to omission of the striatum and midbrain in theories of humor processing.

For our striatal ROI analyses, we found significant activation in the DS and VS for humor comprehension in both the Joke and *Seinfeld* viewing tasks. This supports and extends our initial hypothesis that the DS is involved in humor comprehension. First, the DS is implicated in cognitive functions that underlie humor comprehension, including inhibition of prepotent responses (Zandbelt and Vink, 2010; MacDonald and Monchi, 2011; Akkermans et al., 2018), cognitive flexibility (Crinion et al., 2006; MacDonald and Monchi, 2011; Mestres-Missé et al., 2012), and working memory (Lewis et al., 2004; MacDonald and Monchi, 2011; Darvas et al., 2014). Furthermore, the DS is functionally and structurally connected to frontotemporal cortical regions that have been implicated in humor comprehension and related processes, such as the inferior frontal gyrus (Kireev et al., 2015; Haber, 2016; Graff-Radford et al., 2017). For example, the right putamen demonstrates functional connectivity with the left inferior frontal gyrus, the left superior temporal gyrus, the left precentral gyrus, and the left middle temporal gyrus during language processing (Viñas-Guasch and Wu, 2017), and the left caudate head and the inferior frontal gyrus demonstrate increased functional connectivity during deliberate deception in young, healthy humans (Kireev et al., 2015). Finally, there is evidence that patients with Parkinson's disease, in which the DS is dopamine depleted, experience deficits in humor comprehension but not humor appreciation (M. Prenger, K. Van Hedger, K. Seergobin, AM Owen, PA MacDonald, unpublished observations). Together, this body of literature supports the notion that the DS is intricately involved in social and cognitive functions, such as humor comprehension, via its connections with cortical areas that have a demonstrated role in humor comprehension. Here, we have demonstrated that the DS indeed plays a role in humor comprehension and have replicated this result across two different humor processing elicitation methods.

The involvement of the VS in humor comprehension was somewhat unanticipated. There are a few studies that implicate the VS and the ventral tegmental area (VTA; the region that supplies dopamine to the VS) in humor comprehension (Chan et al., 2012, 2023). It is possible that the VS contributes to humor comprehension by motivating the resolution of incongruities. In their discussion, Chan et al. (2012) suggest that VS activation during humor comprehension might be related to a feeling of relief associated with incongruity resolution that might be separate from the amusement feeling of humor appreciation. The VS is also implicated in reward expectation (Knutson et al., 2001; de la Fuente-Fernández et al., 2002; Filimon et al., 2020; Pool et al., 2022). Given that humor comprehension is an effortful process, activation of the VS in anticipation of a potential humor-appreciation-related reward might help to drive the humor comprehension process forward. This could be related to the role of the VS in humor generation (another effortful process), demonstrated by Amir and Biederman (2016).

Unsurprisingly, we observed significant activation of the VS during humor appreciation in the Joke task and the *Seinfeld* viewing task. Activation of the VS during humor appreciation has been well established in the previous literature (Mobbs et al., 2003; Azim et al., 2005; Mobbs et al., 2005; Watson et al., 2007; Bekinschtein et al., 2011; Neely et al., 2012; Noh et al., 2014; Shibata et al., 2014; Chang et al., 2023), and aligns with the role of the VS in reward processing and prediction error (Schultz, 2016). Importantly, our Bayesian one-sample *t* tests supported the null hypothesis that the DS is not activated during humor appreciation. DS activation during humor processing appears not to be linked to the rewarding nature of humor appreciation. Rather, activation of the DS during humor processing seems related to the cognitive processes that support humor comprehension.

Our findings represent an advancement in the field of humor research by establishing roles for both the DS and VS in humor comprehension and for the VS only in humor appreciation. This could suggest that midbrain dopaminergic signaling is an important component of humor processing. So far, only behavioral research has demonstrated humor comprehension deficits in dopamine-related disorders, such as Parkinson's disease (Benke et al., 1998; Thaler et al., 2012; Mensen et al., 2014). Further research using neuroimaging, clinical cohorts, and pharmacological manipulation would provide further support for the hypothesis that dopamine signaling is involved in humor comprehension and appreciation.
